# Types and Concentrations of Blood-Based Biomarkers in Adults With Peripheral Neuropathies: A Systematic Review and Meta-analysis

**DOI:** 10.1001/jamanetworkopen.2022.48593

**Published:** 2022-12-27

**Authors:** Joel Fundaun, Melissa Kolski, Miguel Molina-Álvarez, Georgios Baskozos, Annina B. Schmid

**Affiliations:** 1 Nuffield Department of Clinical Neurosciences, University of Oxford, Oxford, United Kingdom; 2 Department of Physical Therapy and Human Movement Sciences, Northwestern University, Feinberg School of Medicine, Chicago, Illinois; 3 Musculoskeletal Outpatient Department, Shirley Ryan AbilityLab, Chicago, Illinois; 4 Area of Pharmacology, Nutrition, and Bromatology, Department of Basic Health Sciences, Universidad Rey Juan Carlos, Madrid, Spain

## Abstract

**Importance:**

Peripheral neuropathies are common conditions and can result in numbness, paresthesia, motor deficits, and pain. There is increasing evidence for the use of biomarkers as clinical indicators of the presence, severity, and prognosis of nerve lesions; however, biomarker identification has largely been focused on disorders of the central nervous system, and less is known about their role in the peripheral nervous system.

**Objective:**

To assess blood-based biomarker concentrations associated with nerve involvement in patients with peripheral neuropathy compared with control participants.

**Data Sources:**

Ovid, MEDLINE, Embase, and CINAHL were searched from inception to September 23, 2021.

**Study Selection:**

Observational studies reporting on blood biomarkers in patients diagnosed with peripheral neuropathy were included. This review was preregistered on PROSPERO and followed the Preferred Reporting Items for Systematic Reviews and Meta-analyses (PRISMA) reporting guideline. Data were abstracted by 1 investigator and independently reviewed by a second.

**Data Extraction and Synthesis:**

Data were meta-analyzed when at least 2 studies reported the same biomarker with comparable methodology. Fixed-effects models were used when only 2 studies were included; random-effects models were used when more than 2 studies were included.

**Main Outcomes and Measures:**

The outcome of interest was concentration of biomarkers.

**Results:**

This review included 36 studies reporting on 4414 participants, including 2113 control participants and 2301 patients with peripheral neuropathy with 13 distinct peripheral neuropathy diagnoses. Diabetic neuropathy was the most common neuropathy diagnosis (13 studies), followed by Charcot-Marie-Tooth disease (6 studies) and Guillain-Barre syndrome (6 studies). Overall, 16 different blood-based biomarkers associated with nerve involvement were evaluated. The most used were neurofilament light chain, S100B, brain-derived neurotrophic factor, and neuron-specific enolase. Patients with peripheral neuropathy demonstrated significantly higher levels of neurofilament light chain compared with controls (standardized mean difference [SMD], 0.93 [95% CI, 0.82 to 1.05]; *P* < .001). There were no significant differences in levels of S100B (SMD, 1.10 [95% CI, −3.08 to 5.28]; *P* = .38), brain-derived neurotrophic factor (SMD, −0.52 [95% CI, −2.23 to 1.19]; *P* = .40), or neuron-specific enolase (SMD, −0.00 [95% CI, −1.99 to 1.98]; *P* = .10) in patients with peripheral neuropathy compared with control participants.

**Conclusions and Relevance:**

The findings of this systematic review and meta-analysis support the use of neurofilament light chain as a blood-based measure associated with the presence of neuronal injury in patients with peripheral neuropathy.

## Key Points

**Question:**

Are peripheral neuropathies associated with altered levels of blood-based biomarkers related to nerve involvement?

**Findings:**

This systematic review and meta-analysis included 36 studies reporting on 4414 participants, including 2301 patients with peripheral neuropathy and 2113 controls. Neurofilament light chain was the most commonly reported marker (17 studies) and demonstrated significantly increased serum or plasma concentrations in patients with peripheral neuropathy compared with controls.

**Meaning:**

These findings suggest that a blood-based measure of neurofilament light chain may be a useful indicator of neuronal injury in patients with peripheral neuropathy.

## Teaser

This systematic review and meta-analysis assesses blood-based biomarker concentrations associated with nerve involvement in patients with peripheral neuropathy compared with control participants.

## Introduction

Peripheral neuropathies are common conditions, with an estimated prevalence of up to 7% in the general population^1^ and an increasing global burden with older age.^2^ Patients with peripheral neuropathy often experience significant pain, weakness, and paresthesia that can result in poor balance, limited mobility, increased risk of lower-limb amputation, and shorter mean survival compared with age- and sex-matched controls.^3^ The complex clinical presentations of peripheral neuropathies can lead to delayed or missed diagnosis^4^ and often have limited treatment strategies.^5^

For central neurological conditions, biomarkers have led to significant advances in the diagnosis, prognosis, and management of numerous conditions, including Alzheimer disease,^6^ traumatic brain injury,^7^ multiple sclerosis,^8^ and amyotrophic lateral sclerosis (ALS).^9^ Although biomarker research was initially limited to cerebrospinal fluid (CSF), advanced immunoassays have enabled the identification of blood-based biomarkers.^10^ These blood-based assays enable the detection of biomarkers related to the peripheral nervous system. Contrasting the expanding evidence for their efficacy in the central nervous system, the role of biomarkers in peripheral nerve disorders is currently less well understood. Specifically, the identification and role of peripheral nerve biomarkers in peripheral neuropathies remains unclear.

Therefore, the aim of this review was to assess differences in the concentrations of blood-based biomarkers associated with nerve involvement in patients with peripheral neuropathy compared with control participants.

## Methods

The reporting of this systematic review and meta-analysis followed the updated Preferred Reporting Items for Systematic Reviews and Meta-analyses (PRISMA) reporting guideline.^11^ This study was preregistered on PROSPERO (CRD42021288101).

### Data Sources

We searched Ovid, MEDLINE, Embase, and CINAHL from inception to September 23, 2021, for studies published in English. Search strategies were developed with a medical librarian and are provided in eTable 1 in Supplement 1.

### Study Selection

We included observational studies reporting on a quantitative blood-based measure of a biomarker associated with nerve involvement in patients with peripheral neuropathy vs a comparative control group (healthy or comorbid control group without peripheral neuropathy or central nervous system diagnosis). Participants were considered to have peripheral neuropathy if the study reported the use of established diagnostic criteria or confirmed neurological contributions through a clinical neurological examination, electrodiagnostic, or quantitative sensory testing.

Exclusion criteria included: participants younger than 18 years, participants with a concomitant central nervous system disorder, studies without a control group or only including a comorbid control group with neuropathy, studies that did not include biomarkers associated with nerve involvement, data from nonblood biosamples (eg, urine, CSF, saliva), nonquantitative methods to assess biomarker concentrations (eg, explorative proteomics or Western blot), and case series, conference abstracts, and randomized clinical trials. When studies met all inclusion criteria but included a mixture of pediatric and adult participants, we contacted the study authors to obtain separate biomarker data for the adult population.

Two reviewers (J.F. and M.M.A.) initially screened study eligibility using titles and abstracts, followed by full texts. Disagreements in selection were resolved by discussion or by mediation of a third reviewer (A.B.S.).

### Quality Assessment

Study quality and risk of bias were assessed using the Newcastle-Ottawa Scale (NOS) for observational studies, including cohort, case-control, and cross-sectional study designs. These scales assess selection, comparability, and outcome. Case-control and cohort studies are scored from 0 to 9, with higher scores suggesting lower risk of bias. This score does not include established cutoffs. An adapted NOS for cross-sectional studies^12^ was used, which is scored out of 10 and has recommended cutoff scores of 0 to 3 indicating high risk; 4 to 7, moderate risk; and 8 to 10, low risk. Two independent reviewers assessed each study for risk of bias (J.F. and M.K.). Disagreements between reviewers were resolved through consensus or by mediation of a third reviewer (A.B.S.).

### Biomarker Selection

Only biomarkers associated with nerve involvement were selected for inclusion and analysis. Biomarkers associated with neuropathy but not directly indicating nerve involvement were excluded (eg, cytokines, chemokines). When the neural relationship of a protein biomarker was unclear, we cross-referenced its physiological function and tissue expression using the Human Protein Atlas.^13^ Studies reporting the use of microRNA were screened for neural cell expression using a freely available database reporting expression of animal central nervous system tissue (CNS microRNA profiles; Washington University School of Medicine in St Louis). Neural cell specificity was then cross-referenced with another available RNA database (RNAcentral; European Bioinformatics Institute). We excluded biomarkers whose predominant role and function are outside of the nervous system.

### Data Extraction

Data were extracted into a standardized spreadsheet. Extracted data included study and participant characteristics (study design, participant age and sex, timing of sample collection), criteria and duration of peripheral neuropathy diagnosis; analytical platform for biomarker detection (eg, single molecule array, enzyme-linked immunosorbent assay), biomarker concentration for patients and controls, and biomarker diagnostic accuracy (eg, sensitivity, specificity, positive and negative predictive values). When patient populations were compared with multiple control groups, the healthy control group (without disease) was selected as the comparator.

Biomarker concentrations reported as means and SDs were extracted when possible. Per the *Cochrane Handbook*,^14^ alternative summary statistics were transformed to means and SDs using recommended calculations^15^ or estimated using Plot Digitizer Software^16^ when only reported graphically. Data were extracted by 1 reviewer (J.F.) and independently checked by another reviewer (M.K.). We attempted to contact study authors to obtain any missing or unclear data.

### Statistical Analysis

All statistical calculations were performed in R software version 4.0.3 (R Project for Statistical Computing)^17^ using the packages *meta* and *metafor*.^18^ Data were meta-analyzed when at least 2 studies reported on the same biomarker, even if different assays were used. We used a random-effects model with restricted maximum likelihood and inverse variance weighting methods to analyze biomarker data included from more than 2 studies. The Knapp-Hartung adjustment was used to control for the standard error of the pooled effect. A fixed-effect model was used to meta-analyze biomarker data when only 2 studies were included to properly account for between-study variance.^19^ Effect estimates using standardized mean differences (SMDs) and 95% CIs were calculated for biomarker concentrations using Hedges *g* to correct for bias from small sample sizes. Statistical significance was set at 2-sided *P* < .05. Heterogeneity was calculated using *I*^2^ statistics and interpreted as 0% to 40% indicating might not be important; 30% to 60%, moderate; 50% to 90%, substantial; and 75% to 100%, considerable.^14^

Preplanned subgroup meta-analysis according to type of peripheral neuropathy was performed when 2 or more studies analyzed the same biomarker in a similar diagnosis of peripheral neuropathy. Like the primary analysis, overall effect estimates of the concentration of each biomarker were calculated within each subgroup of peripheral neuropathy compared with controls using SMDs and 95% CIs. In individual studies that compared 2 or more subgroups of patients with peripheral neuropathy with 1 control group, the number of control participants was divided by the number of patient subgroups.^14^ A post hoc analysis of primarily axonal and demyelinating peripheral neuropathy subtypes was performed as described in the eMethods in Supplement 1.

We also preplanned to meta-analyze diagnostic accuracy data from at least 2 studies using the same biomarker. However, these data were not meta-analyzed, as all included studies reporting diagnostic accuracy data used different diagnostic cutoff thresholds and had high between-study heterogeneity. Results from single studies unable to be meta-analyzed were narratively synthesized using the principles of the *Guidance on the Conduct of Narrative Synthesis in Systematic Reviews: A Product from the ESRC Methods Programme*.^20^

## Results

**Figure 1.**
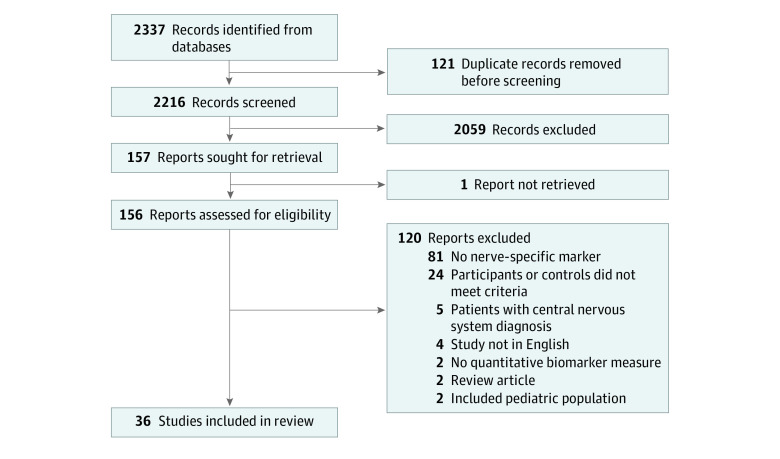
Study Selection Flowchart

We screened 2216 nonduplicated records, resulting in 36 studies^21,22,23,24,25,26,27,28,29,30,31,32,33,34,35,36,37,38,39,40,41,42,43,44,45,46,47,48,49,50,51,52,53,54,55,56^ being included after eligibility assessment, including 2301 patients with peripheral neuropathy and 2113 control participants (Figure 1). Participant characteristics are detailed in eTable 2 in Supplement 1. The most commonly reported type of peripheral neuropathy was diabetic neuropathy (13 studies^21,22,23,24,25,26,27,28,29,30,31,32,33^), followed by Charcot-Marie-Tooth disease (6 studies^34,35,36,37,38,39^), Guillain-Barre Syndrome (6 studies^26,40,41,42,43,44^), chronic inflammatory demyelinating polyneuropathy (5 studies^26,34,45,46,47^), acute inflammatory demyelinating polyneuropathy (3 studies^26,34,45^), and hereditary transthyretin-mediated amyloidosis with polyneuropathy (3 studies^48,49,50^). Single studies included hexane-induced neuropathy,^51^ critical illness polyneuropathy,^52^ acute motor axonal neuropathy,^34^ axonal sensorimotor neuropathy,^26^ vasculitic neuropathy,^53^ rheumatoid arthritis with neuropathy,^54^ leprosy with neuropathy,^55^ and a cohort including various neuropathies^56^ (Table). Data from 3 microRNA biomarker studies were excluded, as the examined markers were not exclusive to the nervous system and involved a diverse range of physiological functions in numerous disease states (eg, cancer, cardiac disease, immune system disorders).

**Table.** Study Characteristics, Including Type of Neuropathy and Concentrations of Blood Biomarkers

Source	Diagnosis	Control type	Biomarker (blood measure, assay)	Concentration, mean (SD)	
				Patient	Control
Afarideh et al,^21^ 2019	Diabetic neuropathy (n = 44)	Healthy (n = 45)	S100B (serum, ELISA)	212.42 (76.79) pg/mL^a^	65.5 (30.59) pg/mL^a^
Altmann et al,^40^ 2020	GBS (n = 27)	Without neuropathy (n = 22)	NFL (serum, SIMOA)	118.9 (141.29) pg/mL^a^	9.07 (3.24) pg/mL^a^
Azoulay et al,^22^ 2020	Diabetic neuropathy (n = 23)	Diabetic without neuropathy (n = 67)	BDNF (serum, ELISA)	14.34 (4.54) ng/mL	18.92 (5.47) ng/mL
Bischof et al,^53^ 2018	Vasculitic neuropathy (n = 11)	Healthy (n = 30)	NFL (serum, SIMOA)	586.91 (784.51) pg/mL	25.62 (11.43) pg/mL^a^
Celikbilek et al,^23^ 2014	Diabetic neuropathy (n = 37)	Healthy (n = 50)	S100B (serum, ELISA)	9.79 (1.75) pg/mL^a^	14.88 (9.16) pg/mL^a^
			GFAP (serum, ELISA)	0 (608.28) ng/mL^a^	0 (707.12) ng/mL^a^
Ghafouri-Fard et al,^45^ 2021	AIDP (n = 22); CIDP (n = 31)	Healthy (n = 49)	BDNF (serum, real-time PCR)	AIDP: −9.77 (1.61)^a^ expression levels	−10.18 (6.35) expression levels
				CIDP: −7.51 (2.09) expression levels	
Frithiof et al,^52^ 2021	Critical illness polyneuropathy (n = 11)	Critical illness without neuropathy (n = 7)	NFL (plasma, SIMOA)	631.43 (545.88) pg/mL^a^	156.83 (135.49) pg/mL^a^
			GFAP (plasma, SIMOA)	181.93 (121.65) pg/mL^a^	90.26 (45.36) pg/mL^a^
			Tau (plasma, SIMOA)	3.67 (4.16) pg/mL^a^	1.27 (0.79) pg/mL^a^
Hayashi et al,^46^ 2021	CIDP (n = 11)	Healthy (n = 7)	NFL (serum, SIMOA)	166.6 (337.8) pg/mL^a^	12.2 (6.3) pg/mL^a^
Jadhav et al,^55^ 2011	Leprosy with neuropathy (n = 48)	Healthy (n = 160)	S100 (serum, ELISA)	56.86 (39.6) AU	28.7 (28.5) AU
Kapoor et al,^48^ 2019	Hereditary transthyretin amyloidosis neuropathy (n = 20)	Healthy (n = 16)	NFL (plasma, SIMOA)	68.4 (71.29) pg/mL	15.45 (7.26) pg/mL
Kim et al,^34^ 2019	AIDP (n = 14); CIDP (n = 36); AMAN (n = 20); CMT (n = 39)	Healthy (n = 20)	p75 neurotrophin receptor (serum, ELISA)	CIDP: 256 (31.26) pg/mL	73.69 (40.58) pg/mL
				AIDP: 207.3 (37.85) pg/mL	
				CMT1a: 12.74 (5.32) pg/mL	
				AMAN: 128.83 (88.76) pg/mL^a^	
			NCAM (serum, ELISA)	CIDP: 4960 (476) pg/mL	2298 (303) pg/mL
				AIDP: 4729 (661) pg/mL	
				CMT1a: 6663 (277) pg/mL	
				AMAN: 3095 (2242.89) pg/mL^a^	
Kortvelyessy et al,^41^ 2020	GBS-high (n = 3); GBS-low (n = 18)	Nonneuropathy (n = 19)	NFL (serum, SIMOA)	GBS-albumin low: 2871.6 (3946.0) pg/mL	50.7 (32.75) pg/mL
				GBS-albumin high: 397.4 (537.9) pg/mL	
Li et al,^24^ 2021	Diabetic neuropathy (n = 56)	Diabetic without neuropathy (n = 24)	GAP-43 (serum, real-time PCR)	0.821 (0.561) expression level	1.064 (0.367) expression level
Li et al,^25^ 2013	Diabetic neuropathy (n = 214)	Healthy (n = 136)	NSE (serum, electrochemiluminescence immunoassay automatic analyzer)	10.8 (2.8) ug/L	8.7 (1.7) ug/L
Lieverloo et al,^47^ 2019	CIDP: induction (n = 29); maintenance (n = 24); remission (n = 27)	Healthy (n = 30)	NFL (serum, SIMOA)	Induction: 47.33 (38.18) pg/mL^a^	24.03 (10.80) pg/mL^a^
				Maintenance: 29.7 (14.06) pg/mL^a^	
				Remission: 30.67 (18.02) pg/mL^a^	
Mariotto et al,^56^ 2020	Various peripheral neuropathies (n = 37)	Healthy (n = 37)	NFL (serum, SIMOA)	22.93 (21.11) pg/mL^a^	6.63 (3.29) pg/,mL^a^
Martin-Aguilar et al,^43^ 2021	GBS (n = 98)	Healthy (n = 53)	NFL (serum, SIMOA)	93.86 (156.28) pg/mL^a^	9.67 (6.42) pg/mL^a^
Mateos-Hernandez et al,^42^ 2016	GBS (n = 8)	Healthy (n = 4)	Piccolo (serum, ELISA)	4.0517 (1.2155) ug/mg	0.0005 (0.0001) ug/mg
Millere et al,^35^ 2021	CMT (n = 83)	Healthy (n = 56)	NFL (plasma, SIMOA)	13.86 (9.81) pg/ml	5.62 (1.97) pg/ml
Xiaowei et al,^51^ 2014	Hexane-induced peripheral neuropathy (n = 18)	Healthy (n = 106)	Human myelin protein P0 (serum, ELISA)	407.21 (93.60) pg/mL^a^	203.04 (86.49) pg/mL^a^
Niezgoda et al,^26^ 2017	Demyelinating polyneuropathy (n = 80); axonal polyneuropathy (n = 40); diabetic polyneuropathy (n = 20)	Healthy (n = 20)	Neural cellular adhesion molecule (serum, ELISA)	Demyelinating: 4588.7 (1763.6) ng/mL	1549.1 (295.4) ng/mL
				Axonal: 3138.4 (1221.3) ng/mL	
				Diabetic: 2869.7 (923.5) ng/mL	
Ozuguz et al,^27^ 2016	Diabetic neuropathy (n = 26)	Nonneurological disease (n = 70)	Nerve growth factor (serum, ELISA)	13.1 (2.6) pg/mL	14.2 (4.7) pg/mL
Rossor et al,^36^ 2016	CMT (n = 90)	Healthy (n = 79)	Neurofilament heavy chain (plasma, ELISA)	27.4 (22.04) ng/mL^a^	Mean: 21.5 (18.64) ng/mL^a^
Maia et al,^49^ 2020	Hereditary transthyretin-mediated amyloidosis with polyneuropathy, group 1 (n = 18); group 2 (n = 26)	Healthy (n = 16)	NFL (plasma, SIMOA)	Group 1: 29.34 (47.37) pg/mL	5.57 (4.44) pg/mL
				Group 2: 117.06 (98.18) pg/mL	
Salih et al,^54^ 2000	Rheumatoid arthritis with peripheral neuropathy (n = 28)	Healthy (n = 28)	Antineuroblastoma cell antibodies (serum, ELISA)	IgG: 4.86 (2.76) AU^a^	IgG: 2.29 (1.58) AU^a^
				IgM: 1.85 (2.49) AU^a^	IgM: 1.06 (1.15) AU^a^
Sandelius et al,^37^ 2018	CMT (n = 75)	Healthy (n = 67)	NFL (plasma, SIMOA)	25.63 (12.07) pg/mL^a^	15.57 (7.48) ng/mL^a^
Sandhu et al,^28^ 2008	Diabetic neuropathy (n = 24)	Healthy (n = 26)	NSE (whole blood, PCR)	0.0067 (0.0038) expression level^a^	0.0094 (0.0048) expression level^a^
Qiao et al,^29^ 2015	Diabetic neuropathy (n = 23)	Nonneurological disease (n = 62)	Neurofilament heavy chain (serum, ELISA)	739.98 (791.28) pg/mL^a^	378.56 (329.28) pg/mL^a^
Sessa et al,^44^ 1997	GBS (n = 61)	Healthy (n = 40)	Myelin-associated β4 integrin (serum, ELISA)	0.208 (0.606)	0.003 (0.020)
Sun et al,^30^ 2018	Diabetic neuropathy (n = 65)	Healthy (n = 110)	Nerve growth factor (serum, ELISA)	42.7(4.9) pg/mL	64.3 (11.7) pg/mL
			BDNF (serum, ELISA)	1739.8 (132.9) pg/mL	2246.7 (331.5) pg/mL
Ticau et al,^50^ 2021	Hereditary transthyretin-mediated amyloidosis with polyneuropathy (n = 159)	Healthy (n = 57)	NFL (plasma, SIMOA)	69.43 (60.50) pg/mL	16.25 (45.87) pg/mL
Wang et al,^38^ 2020	CMT: group 1 (n = 20); group 2 (n = 31)	Healthy: group 1 (n = 20); group 2 (n = 24)	TMPRSS5 (plasma, immuno-PCR, SIMOA)	NPX: group 1: 4.37 (0.62)^a^	NPX control 1: 3.32 (0.50)^a^
				Group 2: 4.42 (0.49)^a^	Control 2: 3.43 (0.648)^a^
			NFL (plasma, immuno-PCR, SIMOA)	NPX: group 1: 3.60 (0.38)^a^	NPX control 1: 2.91 (0.43)^a^
				Group 2: 3.52 (0.76)^a^	Control 2: 2.84 (0.44)^a^
Wang et al,^39^ 2021	CMT, TMRSS5 (n = 65); NFL (n = 41)	Healthy, TMRSS5 (n = 52); NFL (n = 40)	TMPRSS5 (plasma, immuno-PCR, SIMOA)	NPX: 4.63 (0.74)^a^	NPX: 3.57 (0.52)^a^
			NFL (plasma, immuno-PCR, SIMOA)	NPX: 3.5 (0.60)^a^	NPX: 2.83 (0.45)^a^
Ziegler et al,^31^ 2019	Diabetic neuropathy (n = 304)	Healthy (n = 354)	Neurotrophin-3 (serum, inflammation multiplex immunoassay)	NPX: 0.87 (0.40)	NPX: 1.03 (0.34)
Morgenstern et al,^32^ 2021	Diabetic neuropathy (n = 63)	Healthy (n = 30)	NFL (serum, SIMOA)	14.69 (8.5) pg/mL	9.88 (3.72) pg/mL
			Myelin protein zero (serum, SIMOA)	4.189 (2.528) expression levels mRNA	7.720 (2.609) expression levels mRNA
Celikbilek et al,^33^ 2014	Prediabetic neuropathy (n = 22)	Healthy (n = 30)	NFL (serum, real-time PCR)	0.0163 (0.0158) mRNA expression^a^	0.004 (0.009) mRNA expression^a^
			NSE (serum, real-time PCR)	0.209 (0.078) mRNA expression^a^	0.145 (0.204) mRNA expression^a^

Abbreviations: AIDP, acute inflammatory demyelinating polyneuropathy; AMAN, acute motor axonal neuropathy; AU, arbitrary units; BDNF, brain-derived neurotrophic factors; CIDP, chronic inflammatory demyelinating polyneuropathy; CMT, Charcot-Marie-Tooth disease; ELISA, enzyme-linked immunosorbent assay; GAP-43, growth association protein 43; GFAP, glial fibrillary acidic protein; GBS, Guillain-Barre syndrome; Ig, immunoglobin; NCAM, neural cell adhesion molecule; NFL, neurofilament light chain; NSE, neuron-specific enolase; NPX, normalized protein expression; PCR, polymerase chain reaction; SIMOA, single molecule array; TMPRSS5, transmembrane protease serine 5.

^a^ Indicates data were estimated from graphs or figures.

From all studies, we identified 16 different blood-based biomarkers associated with nerve involvement measured in either serum or plasma. Neurofilament light chain (NFL) was the most studied biomarker (17 studies^32,33,35,37,38,39,40,41,43,46,47,48,49,50,52,53,56^), followed by 3 studies each for brain-derived neurotrophic factor,^22,30,45^ S100B,^21,33,55^ and neuron-specific enolase.^25,28,33^ Biomarkers from 2 studies included nerve growth factor,^27,30^ neural cellular adhesion molecule,^26,34^ glial fibrillary acidic protein,^23,52^ transmembrane protease serine 5,^38,39^ and neurofilament heavy chain^29,36^ (Table). Biomarkers that were identified from single studies are listed and summarized in eTable 3 in Supplement 1. Study risk of bias from median (range) NOS scores was 6 (2-8) for case-control studies, 8 (5-9) for cross-sectional studies, and 7 (4-9) for cohort studies (eTable 4 in Supplement 1).

### Biomarker Meta-analyses

**Figure 2.**
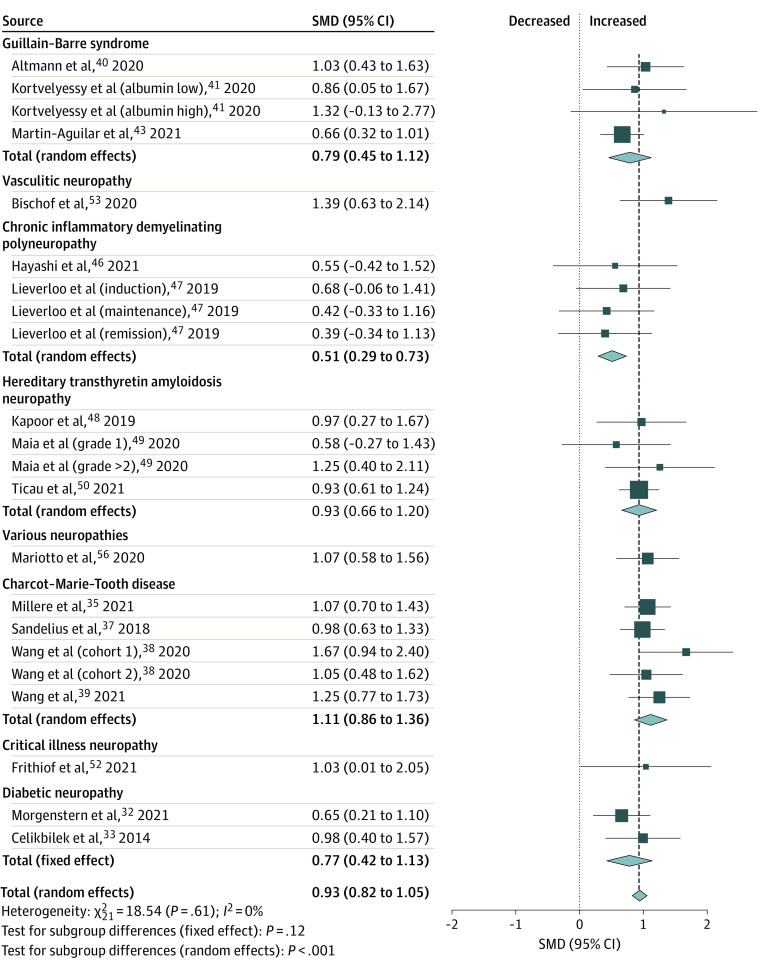
Meta-analysis of Neurofilament Light Chain in Patients With Peripheral Neuropathy Compared With Controls Overall effect sizes, standardized mean differences (SMDs), 95% CIs, and heterogeneity (*I*^2^) are summarized and further subgrouped based on the type of peripheral neuropathy.

**Figure 3.**
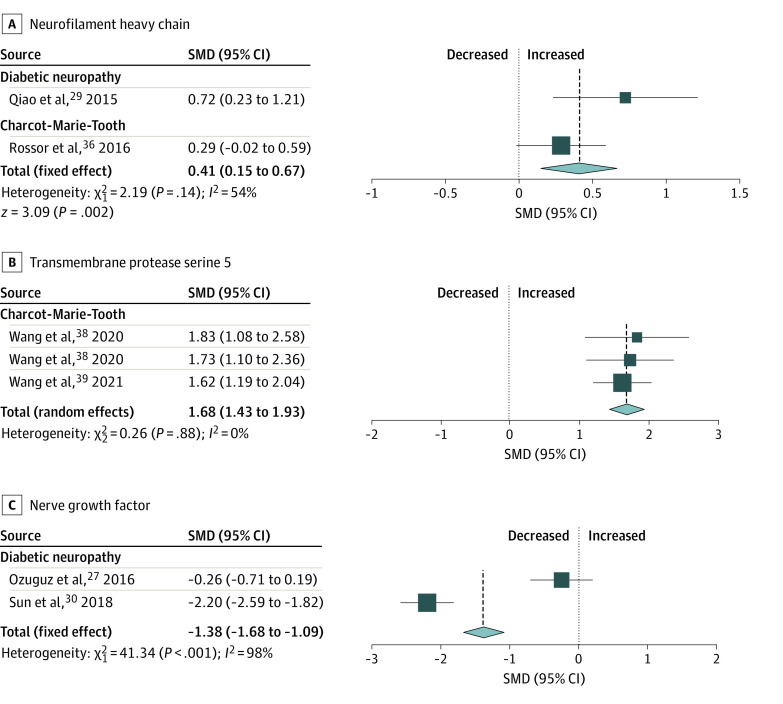
Meta-analyses of Blood-Based Biomarkers That Demonstrated a Statistically Significant Difference of Concentrations in Patients With Peripheral Neuropathy Compared With Controls Overall effect sizes, standardized mean differences (SMDs), 95% CIs, and heterogeneity (*I*^2^) are summarized for each biomarker and further subgrouped based on the type of peripheral neuropathy. The scales for standardized mean differences vary by biomarker.

Blood-based biomarkers that were significantly increased in patients with peripheral neuropathy compared with control participants included NFL (SMD, 0.93 [95% CI, 0.82 to 1.05]; *P* < .001; *I*^2^ = 0%) (Figure 2), neurofilament heavy chain (SMD, 0.41 [95% CI, 0.15 to 0.67]; *P* = .002; *I*^2^ = 54%), and transmembrane protease serine 5 (SMD, 1.68 [95% CI, 1.43 to 1.93]; *P* = .001; *I*^2^ = 0%), with between-study heterogeneity ranging from not important to moderate (Figure 3). In contrast, nerve growth factor was the only biomarker that showed a significant decrease in patients with peripheral neuropathy compared with controls (SMD, −1.38 [95% CI, −1.68 to −1.09]; *P* < .001; *I*^2^ = 98%), with considerable heterogeneity (Figure 3).

**Figure 4.**
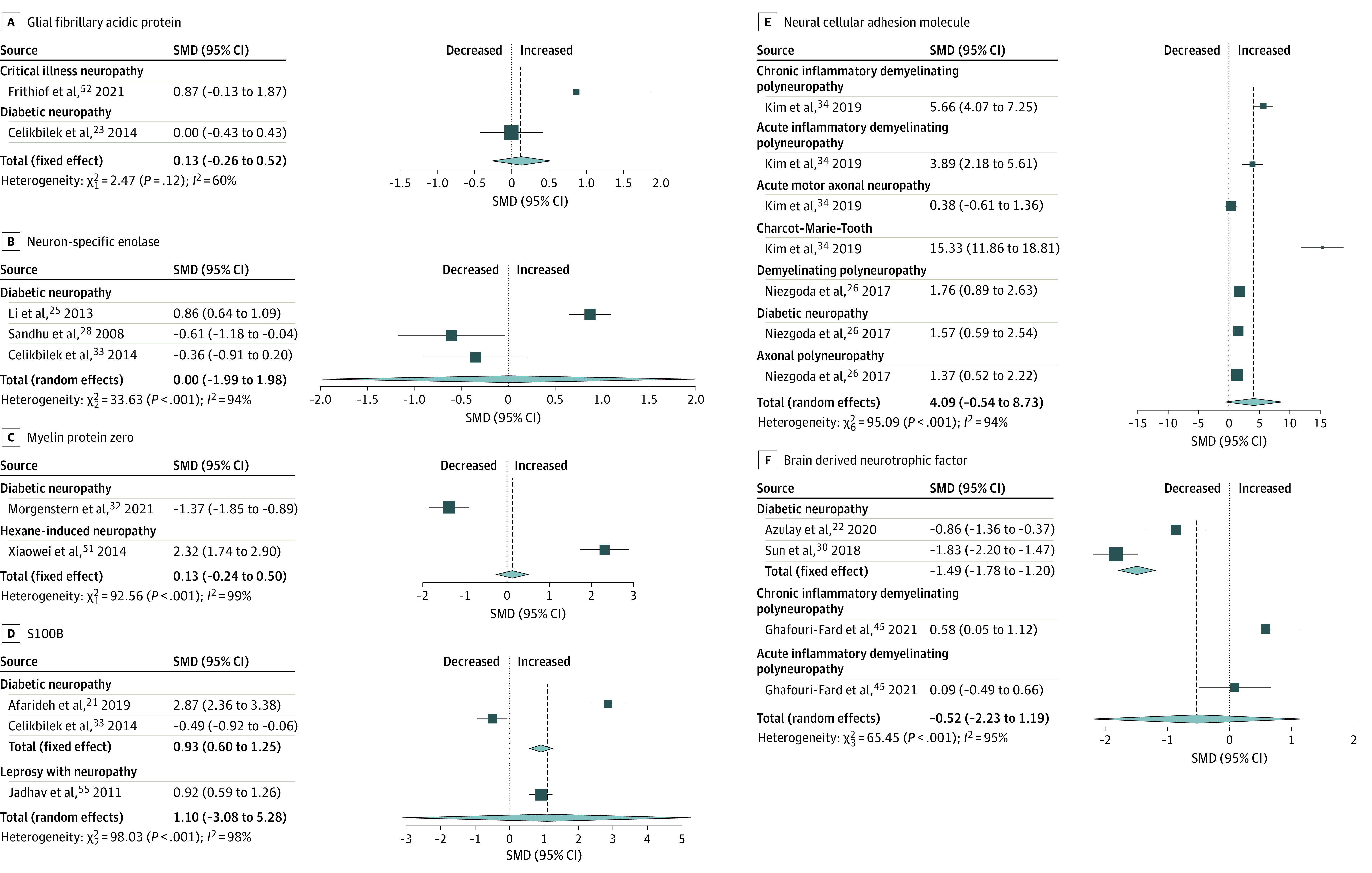
Meta-analyses of Blood-Based Biomarkers That Did Not Demonstrate a Statistically Significant Difference of Concentrations in Patients With Peripheral Neuropathy Compared With Controls Overall effect sizes, standardized mean differences (SMDs), 95% CIs, and heterogeneity (*I*^2^) are summarized for each biomarker and further subgrouped based on the type of peripheral neuropathy. The scales for standardized mean differences vary by biomarker.

Several biomarkers were not significantly different in patients with peripheral neuropathy compared with controls (Figure 4). These included myelin protein zero (SMD, 0.13 [95% CI, −0.24 to 0.50]; *P* = .50; *I*^2^ = 99%), S100B (SMD, 1.10 [95% CI, −3.08 to 5.28]; *P* = .38; *I*^2^ = 98%), brain-derived neurotrophic factor (SMD, −0.52 [95% CI, −2.23 to 1.19]; *P* = .40; *I*^2^ = 95%), glial fibrillary acidic protein (SMD, 0.13 [95% CI, −0.26 to 0.52]; *P* = .50; *I*^2^ = 60%), neural cellular adhesion molecule (SMD, 4.09 [95% CI, −0.54 to 8.73]; *P* = .07; *I*^2^ = 94%), and neuron-specific enolase (SMD −0.00 [95% CI, −1.99 to 1.98]; *P* = .10; *I*^2^ = 94%). All studies had substantial to considerable heterogeneity.

### Diagnostic Subgroup Meta-analyses

When separately analyzed to detect subgroup differences based on type of peripheral neuropathy (Figure 2, Figure 3, and Figure 4), diabetic neuropathy had increased NFL (SMD, 0.77 [95% CI, 0.42 to 1.13]; *P* < .001; *I*^2^ = 0%) and S100B (SMD, 0.93 [95% CI, 0.60 to 1.25]; *P* < .001; *I*^2^ = 99%). Nerve growth factor (SMD, −1.38 [95% CI, −1.68 to −1.09]; *P* < .001; *I*^2^ = 98%) and brain-derived neurotrophic factor (SMD, −1.49 [95% CI, −1.78 to −1.20]; *P* < .001; *I*^2^ = 90%) were decreased. No significant difference was identified in neuron-specific enolase compared with controls (SMD, −0.00 [95% CI, −1.99 to 1.98]; *P* = .10; *I*^2^ = 94%) (Table).

In patients with Guillain-Barre syndrome, there was a significant increase in NFL (SMD, 0.79 [95% CI, 0.45 to 1.12]; *P* = .005; *I*^2^ = 0%). Similarly, patients with chronic inflammatory demyelinating polyneuropathy had significantly increased NFL (SMD, 0.51 [95% CI, 0.29 to 0.73]; *P* = .005; *I*^2^ = 0%). NFL concentrations of predominantly axonal and demyelinating peripheral neuropathy subtypes did not differ (eTable 5 and eFigure 1 in Supplement 1).

Patients with Charcot-Marie-Tooth disease had significantly increased NFL (SMD, 1.11 [95% CI, 0.86 to 1.36]; *P* < .001; *I*^2^ = 0%) and transmembrane protease serine 5 (SMD, 1.68 [95% CI, 1.43 to 1.93]; *P* = .001; *I*^2^ = 0%) compared with controls. Lastly, patients with hereditary transthyretin-mediated amyloidosis with polyneuropathy had significantly increased levels of NFL (SMD, 0.93 [95% CI, 0.66 to 1.20]; *P* = .002; *I*^2^ = 0%) compared with controls.

### Diagnostic Accuracy

Meta-analyses of biomarker diagnostic accuracy could not be performed due to significant differences in concentration cutoff values among studies (eg, NFL range, 8.9 to 155 pg/mL) and varying peripheral neuropathy diagnoses. Biomarker concentration cutoff values and corresponding diagnostic accuracy data are listed in eTable 6 in Supplement 1.

## Discussion

Our systematic review and meta-analysis identified 16 blood-based biomarkers from 36 studies, including 2301 patients with peripheral neuropathy and 2113 control participants. Our meta-analyses identified 4 biomarkers that were significantly altered in patients with peripheral neuropathy compared with controls. Among those, NFL was consistently upregulated in peripheral neuropathy with a large effect size (based on 17 studies^32,33,35,37,38,39,40,41,43,46,47,48,49,50,52,53,56^ and 8 types of neuropathies). Neurofilament heavy chain, transmembrane protease serine 5, and nerve growth factor were also significantly dysregulated, with magnitudes of effect size ranging from moderate to large. However, these results were derived from only 2 studies with higher heterogeneity.

### Association of NFL With Peripheral Neuropathies

Our findings from 854 patients with peripheral neuropathy and 561 controls strongly suggest that a blood-based measure of NFL was a useful biomarker in patients with peripheral neuropathy. Increased concentrations of NFL were consistently identified in each type of peripheral neuropathy using varying methods of blood collection (eg, plasma, serum) and immunoassays (eg, enzyme-linked immunosorbent assay, single molecule array). These findings in peripheral neuropathies are aligned with strong evidence demonstrating increased concentrations of NFL in several central nervous system conditions, including ALS,^6,57^ Alzheimer Disease,^6,58^ multiple sclerosis,^6,8^ and traumatic brain injury.^7,59^ NFL’s functional role in axonal growth and stability and its high expression in neuronal tissue^60^ (eFigure 2 in Supplement 1) make it a robust neuronal biomarker.^61^ Our data corroborate using NFL as a blood-based biomarker not only for central but also for peripheral neuropathies.

### Associations of Additional Biomarkers With Peripheral Neuropathies

Our meta-analyses highlighted additional biomarkers that may be useful in peripheral neuropathies. Neurofilament heavy chain, transmembrane protein serine 5, and nerve growth factor were significantly altered in patients with peripheral neuropathy compared with controls. Biomarkers that were not significantly altered (eg, brain-derived neurotrophic factor, glial fibrillary acidic protein, neural cellular adhesion molecule) may be more limited as blood-based measures of nerve involvement due to their diverse neurological functions.^62,63,64^ However, firm conclusions regarding the use of these biomarkers cannot be drawn, as all meta-analyses (except NFL) were taken from a small number of studies with significant between-study heterogeneity. Further research is needed to understand the role of these biomarkers in the context of different types of peripheral neuropathies.

### Potential Clinical Implications and Considerations for Future Research

#### Early Detection

There is growing evidence for the use of biomarkers in the early detection of nerve involvement. Numerous studies have shown that NFL is a reliable measure of nerve involvement in the presymptomatic stage of central nervous system diseases, including frontotemporal dementia,^65^ Alzheimer disease,^66^ multiple sclerosis,^67^ and ALS.^57^ To date, the role of blood biomarkers for the early or presymptomatic detection of peripheral neuropathy remains understudied. We only identified 1 such study assessing potential early signs of neuropathy in patients with prediabetes.^33^ This study identified increased expression of NFL in patients with prediabetes compared with healthy controls. Therefore, more studies are needed to understand biomarkers’ ability to detect presymptomatic pathology.

#### Diagnosis

Patients with peripheral neuropathy often have comorbid conditions (eg, cardiovascular, immune, or metabolic dysfunction) making it difficult to detect and diagnose neuropathy.^68^ For example, it is currently estimated that physicians only recognize neuropathy symptoms in less than one-third of patients presenting with symptomatic diabetic neuropathy,^69^ supporting the need for improved diagnostic tools. Initial CSF-based diagnostic studies using NFL highlighted its discriminatory ability in multiple neurological conditions.^6^ Advanced immunoassays can now reliably detect lower concentrations of NFL in the blood and strongly correlate with CSF concentrations.^8,70^ Similarly, a 2021 study^58^ using plasma NFL confirmed significant diagnostic implications in multiple neurodegenerative conditions. Although study heterogeneity limited our ability to meta-analyze diagnostic cutoff thresholds, numerous studies in our systematic review demonstrated strong diagnostic accuracy. This included using NFL to discriminate between patients with and without vasculitic neuropathy,^53^ symptomatic vs asymptomatic hereditary transthyretin-mediated amyloidosis with polyneuropathy,^49^ and between patients with Charcot-Marie-Tooth disease and healthy controls.^35,38,39^ Future research considering the optimal type of blood analysis and immunological assay is needed to develop generally accepted clinical diagnostic cutoffs for patients with different types of peripheral neuropathy. Of note, NFL did not seem to be a good marker to differentiate primarily axonal from demyelinating types of neuropathies with elevated levels in both types.

#### Prognosis

Biomarkers could serve important roles in evaluating the severity and prognosis of peripheral neuropathies. Although meta-analyzing this was out of scope for this review, several included studies showed a significant association between NFL and disease severity, including Charcot-Marie-Tooth disease^37,38^ and Guillain-Barre Syndrome.^40,43^ Additionally, 2 studies in patients with diabetic neuropathy identified significant associations between increased NFL concentrations and neuropathic pain,^33^ as well as a hyperalgesic pain phenotype.^32^ Although few studies included long-term follow-up data, 1 study in Guillain-Barre syndrome^43^ highlighted NFL’s ability to estimate patients’ ability to walk or run independently 1 year after disease onset.

#### Treatment Stratification

Biomarkers may also be used to improve treatment through patient stratification. The pathophysiology of neuropathies is diverse and requires unique, individualized treatments. Identifying robust clinical biomarkers could lead to improved quality and cost-effectiveness of care.^71^ Further development and validation is currently required before biomarkers can be used for patient stratification in clinical neurology.^72^ The identification of promising biomarker candidates in this review provides an important initial step to progress toward personalized management for people with peripheral neuropathies.

### Limitations

There are limitations to consider when interpreting our results. First, publication bias may prevent the reporting of negative results of biomarker data. Additionally, only English-language publications were included and between-study heterogeneity in smaller studies (eg, methodological heterogeneity) may limit the generalizability of our findings. The overall quality of included studies was high. Limited reporting or adjustment of confounding variables between participants and controls was the primary limitation, which was only identified in 5 of 36 studies. Furthermore, it is important to consider the pathophysiological differences in peripheral neuropathies. This needs to be remembered when interpreting the overall meta-analyses of certain biomarkers, as some markers may be more useful in certain neuropathies than others.

## Conclusions

In this systematic review and meta-analysis, our findings supported the use of NFL as a blood-based biomarker of nerve involvement in patients with peripheral neuropathy. When compared with other nerve-related biomarkers, NFL was consistently increased in patients with varying types of peripheral neuropathies compared with control participants. Neurofilament heavy chain, transmembrane protease serine 5, and nerve growth factor were also significantly altered in peripheral neuropathy, although these results are based on few studies. Future research is required to assess the temporal patterns, diagnostic accuracy, and prognostic ability of these biomarkers in patients with peripheral neuropathy and peripheral nerve injuries.

## Notes

Supplement 1. **eTable 1.** Search Strategies for All Included Search Engines **eMethods.** Data Extraction and Subtype Selection for Primarily Axonal and Demyelinating Peripheral Neuropathy **eTable 2.** Summary of Diagnostic Criteria and Cohort Characteristics at Study-Level **eTable 3.** Summary of Biomarker Results Reported in Single Studies **eTable 4.** Quality Assessment Using the Newcastle-Ottawa Scales **eTable 5.** Diagnostic Criteria and Neurofilament Light Chain Concentrations Used in the Subgroup Meta-analysis Comparing Primarily Axonal vs Demyelinating Peripheral Neuropathies **eTable 6.** Descriptive Synthesis of Biomarker Diagnostic Accuracy **eFigure 1.** Meta-analysis of Neurofilament Light Chain Comparing Axonal and Demyelinating Subtypes in Patients With Peripheral Neuropathy Compared With Controls **eFigure 2.** Bulk Tissue Gene Expression Profile for Neurofilament Light Chain According to GTEx Portal

Supplement 2.
Data Sharing Statement

## References

[1] Hanewinckel R, van Oijen M, Ikram MA, van Doorn PA. The epidemiology and risk factors of chronic polyneuropathy. Eur J Epidemiol. 2016;31(1):5-20. doi:10.1007/s10654-015-0094-6 26700499 PMC4756033

[2] Elafros MA, Kvalsund MP, Callaghan BC. The global burden of polyneuropathy—in need of an accurate assessment. JAMA Neurol. 2022;79(6):537-538. doi:10.1001/jamaneurol.2022.0565 35404377 PMC9197927

[3] Hoffman EM, Staff NP, Robb JM, St Sauver JL, Dyck PJ, Klein CJ. Impairments and comorbidities of polyneuropathy revealed by population-based analyses. Neurology. 2015;84(16):1644-1651. doi:10.1212/WNL.0000000000001492 25832668 PMC4409579

[4] Brannagan TH III. Current issues in peripheral neuropathy. J Peripher Nerv Syst. 2012;17(suppl 2):1-3. doi:10.1111/j.1529-8027.2012.00387.x 22548615

[5] Koopman RJ, Mainous AG III, Liszka HA, . Evidence of nephropathy and peripheral neuropathy in US adults with undiagnosed diabetes. Ann Fam Med. 2006;4(5):427-432. doi:10.1370/afm.577 17003143 PMC1578655

[6] Bridel C, van Wieringen WN, Zetterberg H, ; NFL Group. Diagnostic value of cerebrospinal fluid neurofilament light protein in neurology: a systematic review and meta-analysis. JAMA Neurol. 2019;76(9):1035-1048. doi:10.1001/jamaneurol.2019.1534 31206160 PMC6580449

[7] Graham NSN, Zimmerman KA, Moro F, . Axonal marker neurofilament light predicts long-term outcomes and progressive neurodegeneration after traumatic brain injury. Sci Transl Med. 2021;13(613):eabg9922. doi:10.1126/scitranslmed.abg9922 34586833

[8] Disanto G, Barro C, Benkert P, ; Swiss Multiple Sclerosis Cohort Study Group. Serum neurofilament light: a biomarker of neuronal damage in multiple sclerosis. Ann Neurol. 2017;81(6):857-870. doi:10.1002/ana.24954 28512753 PMC5519945

[9] Turner MR, Kiernan MC, Leigh PN, Talbot K. Biomarkers in amyotrophic lateral sclerosis. Lancet Neurol. 2009;8(1):94-109. doi:10.1016/S1474-4422(08)70293-X 19081518

[10] Alagaratnam J, von Widekind S, De Francesco D, . Correlation between CSF and blood neurofilament light chain protein: a systematic review and meta-analysis. BMJ Neurol Open. 2021;3(1):e000143. doi:10.1136/bmjno-2021-000143 34223154 PMC8211066

[11] Page MJ, McKenzie JE, Bossuyt PM, . Updating guidance for reporting systematic reviews: development of the PRISMA 2020 statement. J Clin Epidemiol. 2021;134:103-112. doi:10.1016/j.jclinepi.2021.02.003 33577987

[12] van den Berg R, Jongbloed EM, de Schepper EIT, Bierma-Zeinstra SMA, Koes BW, Luijsterburg PAJ. The association between pro-inflammatory biomarkers and nonspecific low back pain: a systematic review. Spine J. 2018;18(11):2140-2151. doi:10.1016/j.spinee.2018.06.349 29960111

[13] Uhlén M, Fagerberg L, Hallström BM, . Proteomics: tissue-based map of the human proteome. Science. 2015;347(6220):1260419. doi:10.1126/science.1260419 25613900

[14] Higgins J, Thomas J, Cumpston M, Li T, Page M, Welch Ve. Cochrane Handbook for Systematic Reviews of Interventions, Version 6.1. The Cochrane Collaboration; 2020.

[15] Wan X, Wang W, Liu J, Tong T. Estimating the sample mean and standard deviation from the sample size, median, range and/or interquartile range. BMC Med Res Methodol. 2014;14(1):135. doi:10.1186/1471-2288-14-135 25524443 PMC4383202

[16] Jelicic Kadic A, Vucic K, Dosenovic S, Sapunar D, Puljak L. Extracting data from figures with software was faster, with higher interrater reliability than manual extraction. J Clin Epidemiol. 2016;74:119-123. doi:10.1016/j.jclinepi.2016.01.002 26780258

[17] R: A Language and Environment for Statistical Computing. R Foundation for Statistical Computing. Accessed November 14, 2022. https://www.R-project.org

[18] Harrer M, Cuijpers P, Furukawa T, Ebert D. Doing Meta-Analysis in R: A Hands-on Guide. CRC Press; 2019.

[19] Borenstein M, Hedges LV, Higgins JP, Rothstein HR. A basic introduction to fixed-effect and random-effects models for meta-analysis. Res Synth Methods. 2010;1(2):97-111. doi:10.1002/jrsm.12 26061376

[20] Popay J, Roberts H, Sowden A, . Guidance on the conduct of narrative synthesis in systematic review: a product from the ESRC Methods Programme. Accessed November 14, 2022. https://www.lancaster.ac.uk/media/lancaster-university/content-assets/documents/fhm/dhr/chir/NSsynthesisguidanceVersion1-April2006.pdf

[21] Afarideh M, Zaker Esteghamati V, Ganji M, . Associations of serum S100B and S100P with the presence and classification of diabetic peripheral neuropathy in adults with type 2 diabetes: a case-cohort study. Can J Diabetes. 2019;43(5):336-344.e2. doi:10.1016/j.jcjd.2019.01.003 30872108

[22] Azoulay D, Abed S, Sfadi A, . Low brain-derived neurotrophic factor protein levels and single-nucleotide polymorphism Val66Met are associated with peripheral neuropathy in type II diabetic patients. Acta Diabetol. 2020;57(7):891-898. doi:10.1007/s00592-020-01508-6 32124075

[23] Celikbilek A, Akyol L, Sabah S, . S100B as a glial cell marker in diabetic peripheral neuropathy. Neurosci Lett. 2014;558:53-57. doi:10.1016/j.neulet.2013.10.067 24211224

[24] Li Y, Ma WG, Li XC. Identification of blood miR-216a, miR-377 and their target genes ANGPTL4, GAP-43 and serum of PPARG as biomarkers for diabetic peripheral neuropathy of type 2 diabetes. Clin Lab. 2021;67(4). doi:10.7754/Clin.Lab.2020.191220 33865244

[25] Li J, Zhang H, Xie M, Yan L, Chen J, Wang H. NSE, a potential biomarker, is closely connected to diabetic peripheral neuropathy. Diabetes Care. 2013;36(11):3405-3410. doi:10.2337/dc13-0590 23846809 PMC3816869

[26] Niezgoda A, Michalak S, Losy J, Kalinowska-Łyszczarz A, Kozubski W. sNCAM as a specific marker of peripheral demyelination. Immunol Lett. 2017;185:93-97. doi:10.1016/j.imlet.2017.03.011 28336415

[27] Ozuguz U, Oruc S, Ulu MS, . Does vitamin D have any role in the improvement of diabetic peripheral neuropathy in type 1 diabetic patients? J Endocrinol Invest. 2016;39(12):1411-1417. doi:10.1007/s40618-016-0509-6 27436228

[28] Sandhu HS, Butt AN, Powrie J, Swaminathan R. Measurement of circulating neuron-specific enolase mRNA in diabetes mellitus. Ann N Y Acad Sci. 2008;1137:258-263. doi:10.1196/annals.1448.044 18837957

[29] Qiao X, Zhang S, Zhao W, . Serum phosphorylated neurofilament-heavy chain, a potential biomarker, is associated with peripheral neuropathy in patients with type 2 diabetes. Medicine (Baltimore). 2015;94(44):e1908. doi:10.1097/MD.0000000000001908 26554790 PMC4915891

[30] Sun Q, Tang DD, Yin EG, . Diagnostic significance of serum levels of nerve growth factor and brain derived neurotrophic factor in diabetic peripheral neuropathy. Med Sci Monit. 2018;24:5943-5950. doi:10.12659/MSM.909449 30145601 PMC6122271

[31] Ziegler D, Strom A, Bönhof GJ, . Deficits in systemic biomarkers of neuroinflammation and growth factors promoting nerve regeneration in patients with type 2 diabetes and polyneuropathy. BMJ Open Diabetes Res Care. 2019;7(1):e000752. doi:10.1136/bmjdrc-2019-000752 31803481 PMC6887496

[32] Morgenstern J, Groener JB, Jende JME, . Neuron-specific biomarkers predict hypo- and hyperalgesia in individuals with diabetic peripheral neuropathy. Diabetologia. 2021;64(12):2843-2855. doi:10.1007/s00125-021-05557-6 34480211 PMC8563617

[33] Celikbilek A, Tanik N, Sabah S, . Elevated neurofilament light chain (NFL) mRNA levels in prediabetic peripheral neuropathy. Mol Biol Rep. 2014;41(6):4017-4022. doi:10.1007/s11033-014-3270-y 24733614

[34] Kim YH, Kim YH, Shin YK, . p75 and neural cell adhesion molecule 1 can identify pathologic Schwann cells in peripheral neuropathies. Ann Clin Transl Neurol. 2019;6(7):1292-1301. doi:10.1002/acn3.50828 31353867 PMC6649441

[35] Millere E, Rots D, Simrén J, . Plasma neurofilament light chain as a potential biomarker in Charcot-Marie-Tooth disease. Eur J Neurol. 2021;28(3):974-981. doi:10.1111/ene.14689 33340200

[36] Rossor AM, Lu CH, Petzold A, . Plasma neurofilament heavy chain is not a useful biomarker in Charcot-Marie-Tooth disease. Muscle Nerve. 2016;53(6):972-975. doi:10.1002/mus.25124 27015106

[37] Sandelius Å, Zetterberg H, Blennow K, . Plasma neurofilament light chain concentration in the inherited peripheral neuropathies. Neurology. 2018;90(6):e518-e524. doi:10.1212/WNL.0000000000004932 29321234 PMC5818017

[38] Wang H, Davison M, Wang K, . Transmembrane protease serine 5: a novel Schwann cell plasma marker for CMT1A. Ann Clin Transl Neurol. 2020;7(1):69-82. doi:10.1002/acn3.50965 31833243 PMC6952315

[39] Wang H, Davison M, Wang K, . MicroRNAs as biomarkers of Charcot-Marie-Tooth disease type 1A. Neurology. 2021;97(5):e489-e500. doi:10.1212/WNL.0000000000012266 34031204 PMC8356381

[40] Altmann P, De Simoni D, Kaider A, . Increased serum neurofilament light chain concentration indicates poor outcome in Guillain-Barré syndrome. J Neuroinflammation. 2020;17(1):86. doi:10.1186/s12974-020-01737-0 32183837 PMC7079539

[41] Körtvelyessy P, Kuhle J, Düzel E, . Ratio and index of neurofilament light chain indicate its origin in Guillain-Barré Syndrome. Ann Clin Transl Neurol. 2020;7(11):2213-2220. doi:10.1002/acn3.51207 33030817 PMC7664266

[42] Mateos-Hernández L, Villar M, Doncel-Pérez E, . Quantitative proteomics reveals Piccolo as a candidate serological correlate of recovery from Guillain-Barré syndrome. Oncotarget. 2016;7(46):74582-74591. doi:10.18632/oncotarget.12789 27776345 PMC5342688

[43] Martín-Aguilar L, Camps-Renom P, Lleixà C, . Serum neurofilament light chain predicts long-term prognosis in Guillain-Barré syndrome patients. J Neurol Neurosurg Psychiatry. 2021;92:70-77. doi:10.1136/jnnp-2020-323899 33154183

[44] Sessa G, Nemni R, Canal N, Marchisio PC. Circulating fragments of myelin-associated alpha 6 beta 4 integrin in Guillain-Barré syndrome. J Neuroimmunol. 1997;80(1-2):115-120. doi:10.1016/S0165-5728(97)00143-4 9413266

[45] Ghafouri-Fard S, Mazdeh M, Nicknafs F, Nazer N, Sayad A, Taheri M. Expression analysis of BDNF, BACE1 and their antisense transcripts in inflammatory demyelinating polyradiculoneuropathy. Mult Scler Relat Disord. 2021;47:102613. doi:10.1016/j.msard.2020.102613 33160139

[46] Hayashi T, Nukui T, Piao JL, . Serum neurofilament light chain in chronic inflammatory demyelinating polyneuropathy. Brain Behav. 2021;11(5):e02084. doi:10.1002/brb3.2084 33617139 PMC8119854

[47] van Lieverloo GGA, Wieske L, Verhamme C, . Serum neurofilament light chain in chronic inflammatory demyelinating polyneuropathy. J Peripher Nerv Syst. 2019;24(2):187-194. doi:10.1111/jns.12319 30973667

[48] Kapoor M, Foiani M, Heslegrave A, . Plasma neurofilament light chain concentration is increased and correlates with the severity of neuropathy in hereditary transthyretin amyloidosis. J Peripher Nerv Syst. 2019;24(4):314-319. doi:10.1111/jns.12350 31583784

[49] Maia LF, Maceski A, Conceição I, . Plasma neurofilament light chain: an early biomarker for hereditary ATTR amyloid polyneuropathy. Amyloid. 2020;27(2):97-102. doi:10.1080/13506129.2019.1708716 31906707

[50] Ticau S, Sridharan GV, Tsour S, . Neurofilament light chain as a biomarker of hereditary transthyretin-mediated amyloidosis. Neurology. 2021;96(3):e412-e422. doi:10.1212/WNL.0000000000011090 33087494 PMC7884985

[51] Jia X, Liu Q, Zhang Y, . Myelin protein zero and its antibody in serum as biomarkers of n-hexane-induced peripheral neuropathy and neurotoxicity effects. Chin Med J (Engl). 2014;127(8):1536-1540. 24762602

[52] Frithiof R, Rostami E, Kumlien E, . Critical illness polyneuropathy, myopathy and neuronal biomarkers in COVID-19 patients: a prospective study. Clin Neurophysiol. 2021;132(7):1733-1740. doi:10.1016/j.clinph.2021.03.016 33875374 PMC8012169

[53] Bischof A, Manigold T, Barro C, . Serum neurofilament light chain: a biomarker of neuronal injury in vasculitic neuropathy. Ann Rheum Dis. 2018;77(7):1093-1094. doi:10.1136/annrheumdis-2017-212045 28743789

[54] Salih AM, Nixon NB, Dawes PT, Mattey DL. Antibodies to neuroblastoma cells in rheumatoid arthritis: a potential marker for neuropathy. Clin Exp Rheumatol. 2000;18(1):23-30. doi:10.1046/j.1529-8027.2000.absjun-6.x 10728440

[55] Jadhav R, Suneetha L, Kamble R, . Analysis of antibody and cytokine markers for leprosy nerve damage and reactions in the INFIR cohort in India. PLoS Negl Trop Dis. 2011;5(3):e977. doi:10.1371/journal.pntd.0000977 21408123 PMC3050910

[56] Mariotto S, Carta S, Bozzetti S, . Sural nerve biopsy: current role and comparison with serum neurofilament light chain levels. J Neurol. 2020;267(10):2881-2887. doi:10.1007/s00415-020-09949-3 32462349

[57] Benatar M, Wuu J, Andersen PM, Lombardi V, Malaspina A. Neurofilament light: a candidate biomarker of presymptomatic amyotrophic lateral sclerosis and phenoconversion. Ann Neurol. 2018;84(1):130-139. doi:10.1002/ana.25276 30014505 PMC11348288

[58] Ashton NJ, Janelidze S, Al Khleifat A, . A multicentre validation study of the diagnostic value of plasma neurofilament light. Nat Commun. 2021;12(1):3400. doi:10.1038/s41467-021-23620-z 34099648 PMC8185001

[59] Gao W, Zhang Z, Lv X, . Neurofilament light chain level in traumatic brain injury: a system review and meta-analysis. Medicine (Baltimore). 2020;99(38):e22363. doi:10.1097/MD.0000000000022363 32957411 PMC7505327

[60] Aguet F, Anand S, Ardlie KG, ; GTEx Consortium. The GTEx Consortium atlas of genetic regulatory effects across human tissues. Science. 2020;369(6509):1318-1330. doi:10.1126/science.aaz1776 32913098 PMC7737656

[61] Khalil M, Teunissen CE, Otto M, . Neurofilaments as biomarkers in neurological disorders. Nat Rev Neurol. 2018;14(10):577-589. doi:10.1038/s41582-018-0058-z 30171200

[62] Bathina S, Das UN. Brain-derived neurotrophic factor and its clinical implications. Arch Med Sci. 2015;11(6):1164-1178. doi:10.5114/aoms.2015.56342 26788077 PMC4697050

[63] Yang Z, Wang KK. Glial fibrillary acidic protein: from intermediate filament assembly and gliosis to neurobiomarker. Trends Neurosci. 2015;38(6):364-374. doi:10.1016/j.tins.2015.04.003 25975510 PMC4559283

[64] Rutishauser U, Acheson A, Hall AK, Mann DM, Sunshine J. The neural cell adhesion molecule (NCAM) as a regulator of cell-cell interactions. Science. 1988;240(4848):53-57. doi:10.1126/science.3281256 3281256

[65] van der Ende EL, Meeter LH, Poos JM, ; Genetic Frontotemporal dementia Initiative (GENFI). Serum neurofilament light chain in genetic frontotemporal dementia: a longitudinal, multicentre cohort study. Lancet Neurol. 2019;18(12):1103-1111. doi:10.1016/S1474-4422(19)30354-0 31701893

[66] Weston PSJ, Poole T, O’Connor A, . Longitudinal measurement of serum neurofilament light in presymptomatic familial Alzheimer’s disease. Alzheimers Res Ther. 2019;11(1):19. doi:10.1186/s13195-019-0472-5 30786919 PMC6383280

[67] Bjornevik K, Munger KL, Cortese M, . Serum neurofilament light chain levels in patients with presymptomatic multiple sclerosis. JAMA Neurol. 2020;77(1):58-64. doi:10.1001/jamaneurol.2019.3238 31515562 PMC6745051

[68] Hughes RAC. Peripheral neuropathy. BMJ. 2002;324(7335):466-469. doi:10.1136/bmj.324.7335.466 11859051 PMC1122393

[69] International Diabetes Federation. IDF Diabetes Atlas. 9th ed. International Diabetes Federation; 2019.

[70] Lu CH, Macdonald-Wallis C, Gray E, . Neurofilament light chain: a prognostic biomarker in amyotrophic lateral sclerosis. Neurology. 2015;84(22):2247-2257. doi:10.1212/WNL.0000000000001642 25934855 PMC4456658

[71] Trusheim MR, Berndt ER, Douglas FL. Stratified medicine: strategic and economic implications of combining drugs and clinical biomarkers. Nat Rev Drug Discov. 2007;6(4):287-293. doi:10.1038/nrd2251 17380152

[72] Matthews PM, Edison P, Geraghty OC, Johnson MR. The emerging agenda of stratified medicine in neurology. Nat Rev Neurol. 2014;10(1):15-26. doi:10.1038/nrneurol.2013.245 24323053

