# Supplementary material for: Types and Concentrations of Blood-Based Biomarkers in Adults With Peripheral Neuropathies: A Systematic Review and Meta-analysis
Source: JAMA Netw Open. 2022 Dec 27;5(12):e2248593. doi: 10.1001/jamanetworkopen.2022.48593 (PMC9857490; doi:10.1001/jamanetworkopen.2022.48593)
Supplement: Supplement 1. — eTable 1. Search Strategies for All Included Search Engines eMethods. Data Extraction and Subtype Selection for Primarily Axonal and Demyelinating Peripheral Neuropathy eTable 2. Summary of Diagnostic Criteria and Cohort Characteristics at Study-Level eTable 3. Summary of Biomarker Results Reported in Single Studies eTable 4. Quality Assessment Using the Newcastle-Ottawa Scales eTable 5. Diagnostic Criteria and Neurofilament Light Chain Concentrations Used in the Subgroup Meta-analysis Comparing Primarily Axonal vs Demyelinating Peripheral Neuropathies eTable 6. Descriptive Synthesis of Biomarker Diagnostic Accuracy eFigure 1. Meta-analysis of Neurofilament Light Chain Comparing Axonal and Demyelinating Subtypes in Patients With Peripheral Neuropathy Compared With Controls eFigure 2. Bulk Tissue Gene Expression Profile for Neurofilament Light Chain According to GTEx Portal [file jamanetwopen-e2248593-s001.pdf]

Supplemental Online Content

Fundaun J, Kolski M, Molina-Álvarez M, Baskozos G, Schmid AB. Types and concentrations of blood-based biomarkers in adults with peripheral neuropathies: a systematic review and meta-analysis. *JAMA Netw Open*. 2022;5(12):e2248593. doi:10.1001/jamanetworkopen.2022.48593

eTable 1. Search Strategies for All Included Search Engines
eMethods. Data Extraction and Subtype Selection for Primarily Axonal and Demyelinating Peripheral Neuropathy
eTable 2. Summary of Diagnostic Criteria and Cohort Characteristics at Study-Level
eTable 3. Summary of Biomarker Results Reported in Single Studies
eTable 4. Quality Assessment Using the Newcastle-Ottawa Scales
eTable 5. Diagnostic Criteria and Neurofilament Light Chain Concentrations Used in the Subgroup Meta-analysis Comparing Primarily Axonal vs Demyelinating Peripheral Neuropathies
eTable 6. Descriptive Synthesis of Biomarker Diagnostic Accuracy
eFigure 1. Meta-analysis of Neurofilament Light Chain Comparing Axonal and Demyelinating Subtypes in Patients With Peripheral Neuropathy Compared With Controls
eFigure 2. Bulk Tissue Gene Expression Profile for Neurofilament Light Chain According to GTEx Portal

This supplemental material has been provided by the authors to give readers additional information about their work.

© 2022 Fundaun J et al. *JAMA Network Open.*

eTable 1. Search Strategies for All Included Search Engines

Medline

1.	“Peripheral Nervous System Diseases"[Mesh] OR "Autoimmune Diseases of the Nervous System"[Mesh] OR "peripheral neuropathy" OR "peripheral nerve disorder" OR "peripheral nervous system" OR "peripheral nerve"
2.	(("blood"[Subheading] OR "blood"[All Fields] OR "blood"[MeSH Terms]) AND ("biomarkers"[MeSH Terms] OR "biomarkers"[All Fields] OR "biomarker"[All Fields])) OR "Biomarkers"[Mesh] OR "biomarker" OR "biologic marker" OR “biological marker” OR "blood biomarker" OR "serum biomarker"
3.	Cohort studies OR Case-control studies OR observational study OR Cross-sectional studies
4.	"Central Nervous System Diseases"[Mesh] OR "Cranial Nerve Diseases"[Mesh] OR "Multiple Sclerosis"[Mesh]
5.	1 AND 2 AND 3 NOT 4

Ovid and Embase

1.	Exp Peripheral Nervous System Diseases/ OR peripheral nerve disorder* OR peripheral nerve disease* OR peripheral neuropath* OR poly neuropath* OR neuralgia OR peripheral nerve injur*
2.	Exp Biomarkers/bl OR (Blood/ and Biomarker/) or biomark* or biologic* marker* or blood biomarker* or serum biomarker*
3.	Exp Central Nervous System Diseases/ OR exp Cranial Nerve Diseases/ OR exp Multiple Sclerosis/
4.	1 AND 2 NOT 3

Cinahl

1.	Exp Peripheral Nervous System Diseases/ OR peripheral nerve disorder* OR peripheral nerve disease* OR peripheral neuropath* OR poly neuropath* OR neuralgia OR peripheral nerve injur*
2.	Exp Biomarkers/bl OR (Blood/ and Biomarker/) or biomark* or biologic* marker* or blood biomarker* or serum biomarker*
3.	*Exp Central Nervous System Diseases/ OR exp Cranial Nerve Diseases/ OR exp Multiple Sclerosis/*
4.	1 AND 2 NOT 3

© 2022 Fundaun J et al. *JAMA Network Open.*

eMethods. Data Extraction and Subtype Selection for Primarily Axonal and Demyelinating Peripheral Neuropathy

All included articles were assessed for distinct diagnostic classification of primarily axonal or demyelinating peripheral neuropathy subtypes. Subtype analysis was not performed if an article included peripheral neuropathy subtypes that were considered to be mixed (without separate group data provided), uncertain, or equivocal diagnostic classifications. As reported in the main-text methods, data extraction (section 2.5) and meta-analysis (section 2.6) were performed if two or more studies reported data of the same primary subtype (axonal or demyelinating) and the same biomarker.

Based on these criteria, the only biomarker that could be meta-analysed was neurofilament light chain. eTable 5 includes the concentrations and varying diagnostic criteria used to distinguish primarily axonal and demyelinating subtypes for each study. Results are presented in eFigure 1.

© 2022 Fundaun J et al. *JAMA Network Open.*

eTable 2. Summary of Diagnostic Criteria and Cohort Characteristics at Study-Level

Source	Diagnosis	Diagnostic criteria (neuropathy group)	Mean duration of diagnosis at sample collection (neuropathy group)	Participant characteristics (mean age, number female)	Control characteristics (mean age, number female)
**Afarideh et al,[21] 2019**	Diabetic neuropathy	NCV, EMG, h-reflex, strength, sensation (Mythili exam score)	10 years	56.5 years, n=17	38.1 years, n=45
**Altmann et al,[40] 2020**	GBS	Neurologic examination, Brighton criteria (levels 1 or 2), NCV	Within 5 days of hospitalisation	55 years, NA	36 years, NA
**Azoulay et al,[22] 2020**	Diabetic neuropathy	Fasting glucose levels, NCV, reduced neuropathy score, small nerve fibre testing	10.4 years	60.5 years, n=34 (total cohort)	60.5 years, n=34 (total cohort)
**Bischof et al,[53] 2018**	Vasculitic neuropathy	Neurologic examination and symptom score, peripheral nerve biopsy, clinical presentation	At time of diagnosis	57.3 years, n=7	NA (age matched controls)
**Celikbilek et al,[23] 2014**	Diabetic neuropathy	Fasting glucose, NCV, EMG	NA	56.2 years, n=50 (full diabetic cohort)	58.9, n=34
**Ghafouri-Fard et al,[45] 2021**	AIDP and CIDP	Clinical examination, electrophysiology, CSF, NINDS classification	NA	36.2 years, n=6 AIDP, n=11 CIDP	35.3 years, n=11
**Frithiof et al,[52] 2021**	Critical illness polyneuropathy	Critical Ilness Neuropathy guidelines (Bolton 2005, Lacomis 2000), EMG, NCV	>11 days from admission	64 years (median), n=0	51 years (median), n=1
**Hayashi et al,[46] 2021**	CIDP	EFNS/PNS 2010 criteria	16.0 weeks (median)	59.6 years (median), n=6	58.0 years (median), n=3
**Jadhav et al,[55] 2011**	Leprosy with neuropathy	Ridley-Jopling classification, strength testing, monofilament testing	NA	NA	NA
**Kapoor et al,[48] 2019**	Hereditary transthyretin amyloidosis neuropathy	Genetic testing, Rasch modified CMT examination, neuropathy impairment score	NA	65.8 years (median), n=8	67 years (median), n=1

© 2022 Fundaun J et al. *JAMA Network Open.*

**Kim et al,[34] 2019**	AIDP, CIDP, AMAN, CMT	EFNS/PNS 2010 criteria; GBS classification criteria (Wakerley 2014; Ho 1995).	NA	NA, AIDP n=3, CIDP n=10, AMAN n=7, CMT, n=17	NA, n=14
**Kortvelyessy et al,[41] 2020**	GBS-high<br><br>GBS-low	Presenting with at least bilateral or flaccid weakness of limbs accompanied by decreased or absent tendon reflexes, CSF cell count under 50 cells/µl, monophasic course and time between onset-nadir of 12 h to 28 days, Brighton collaboration criteria 1 and 2	NA	GBS-high 58.4 years, n=0;<br><br>GBS-low 60.7 years, n=6	64.7 years, n=9
**Li et al,[24] 2021**	Diabetic neuropathy	ADA diagnosis criteria for diabetes, neurologic exam, NCV	9.9 years	61.6 years, n=25	50.1 years, n=13
**Li et al,[25] 2013**	Diabetic neuropathy	WHO classification of diabetes, ADA diabetic neuropathy, neurologic examination, QST, NCV	7.3 years	52.3 years (median), n=70	52.5 (median), n=47
**Lieverloo et al,[47] 2019**	CIDP – Induction<br><br>Maintenance<br><br>Remission	EFNS/PNS 2010 criteria, neurologic exam, NCV	Induction: 22 months (median)<br><br>Maintenance: 71 months (median)<br><br>Remission: 39 months in remission (median)	Induction 61 years, n=8<br><br>Maintenance 59 years, n=7<br><br>Remission 63 years, n=4	62 years, n=11
**Mariotto et al,[56] 2020**	Various peripheral neuropathies	Neurologic examination, sural nerve biopsy, neurophysiological evaluation	NA	65.1 years, n=12	NA
**Martin-Aguilar et al,[43] 2021**	GBS	NINDS classification, Brighton diagnostic criteria, NCV	4 days (median time from symptom onset)	57.4 years, n=42	53.1 years, n=30
**Mateos-Hernandez**	GBS	Clinical examination	NA	57 years, n=4	37.3 years, n=3

© 2022 Fundaun J et al. *JAMA Network Open.*

**et al,[42] 2016**					
**Millere et al,35 2021**	CMT	Clinical/neurophysiological examination or positive genetic testing for a known pathogenic variant in the family, neurography, CMT Neuropathy Score	NA	38.6 years, n=51	35.7 years, n=41
**Xiaowei et al,[51] 2014**	Hexane-induced peripheral neuropathy	Diagnostic Criteria of Occupational Chronic n-hexane Poisoning (GBZ84-2002)	NA	28.6 years, n=18	26.6 years, n=106 (total cohort)
**Niezgoda et al,[26] 2017**	Demyelinating, axonal, and diabetic polyneuropathy	GBS from lab and electrophysiological testing, clinical assessment (overall neuropathy limitations scale), criteria type by EFNS/PNS 2010	NA	Demyelinating 39.3 years, n=30<br><br>Axonal polyneuropathy 49.5 years, n=19<br><br>Diabetic polyneuropathy 46.8 years, n=10	40.3 years, n=10
**Ozuguz et al,[27] 2016**	Diabetic neuropathy	MNSI, vibration sensation, ankle reflexes, monofilament testing (10g)	18.1 years	38 years, n=14	28 years, n=39
**Rossor et al,[36] 2016**	CMT	Genetically confirmed diagnosis of CMT, CMT Examination Score (CMTES, 2nd version)	NA	44 years, n=44	NA, n=23
**Maia et al,[49] 2020**	Hereditary transthyretin-mediated amyloidosis with polyneuropathy	Genetic testing, neurologic examination, polyneuropathy disability score	Group 1 - PND1: 1.4 years, PND 2 and 3: 4.1 years<br><br>Group 2 - PND 1: 2.0 years, PND 2 and 3: 3.9 years, PND 4: 5.4 years	Group 1 – PND 1: 41.0 years, n=7, PND: 2 and 3: 53.5 years,n=8<br><br>Group 2 -PND 1: 57.4, n=1, PND 2 and 3: 62.0 years, n=4, PND 4: 63.8 years , n=1	33.5 years, n=7

© 2022 Fundaun J et al. *JAMA Network Open.*

**Salih et al,[54] 2000**	Rheumatoid arthritis with peripheral neuropathy	American College of Rheumatology revised criteria (1987), NCV, EMG, neurologic symptom and disability scores	11.4 years	64 years, n=18	61 years, n=22
**Sandelius et al,[37] 2018**	CMT	Genetically confirmed CMT, NCV	NA	46.2 years, n=39	47.0 years, n=46
**Sandhu et al,[28] 2008**	Diabetic neuropathy	Clinical examination	18.1 years	59.6 years, n=6	44.2 years, n=8
**Qiao et al,[29] 2015**	Diabetic neuropathy	WHO diabetes diagnosis critiera, neuropathy symptom and disability scores, clinical examination, QST, NCV, EMG	11.0 years (median)	61.6 years, n=15	64.5 years, n=33
**Sessa et al,[44] 1997**	GBS	GBS diagnostic criteria (Asbury and Cornblath, 1990)	2–7 days: n=36 patients,<br><br>8–14 days: n=19 patients,<br><br>15–21 days: n=6 patients	NA	NA
**Sun et al,[30] 2018**	Diabetic neuropathy	WHO and ADA diabetes classification, clinical examination, NCV, EMG	12.7 years	58.2 years, n=35	57.3 years, n=63
**Ticau et al,[50] 2021**	Hereditary transthyretin-mediated amyloidosis with polyneuropathy	Neuropathy Impairment Score 5 -130, polyneuropathy disability score ≤IIIb	NA	60.5 years, n=47	58.6 years, n=15
**Wang et al,[38] 2020**	CMT	Clinical examination, NCV, confirmatory genetic testing, Rasch modified CMT Neuropathy and Examination Scores	NA	Group 1: 47.4 years<br><br>Group 2: 42.1 years, n=32 (total)	Group 1: 47.1 years<br><br>Group 2: 49.4 years, n= 18 (total cohort)
**Wang et al,[39] 2021**	CMT	Clinical examination, NCV, confirmatory genetic testing, Rasch modified	NA	46.7 years, n=61 (total cohort)	46.9 years, n=33

© 2022 Fundaun J et al. *JAMA Network Open.*

		CMT Neuropathy and Examination Scores			
**Ziegler et al,[31] 2019**	Diabetic neuropathy	ADA criteria, electrophysiological testing, QST, skin biopsies, NCV	13.5 years	68.0 years, n=228	68.9 years, n=313
**Morgenstern et al,[32] 2021**	Diabetic neuropathy	German Diabetes Association criteria, neuropathy deficit score, neuropathy symptom score, QST, EMG	11.9 years	62.6 years, n=23	58.0 years, n=16
**Celikbilek et al,[33] 2014**	Pre-diabetic neuropathy	Fasting glucose as defined by ADA, sensory and motor nerve conduction studies, score of ≥4 on the Douleur Neuropathique 4 questionnaire	NA	54.1 years, n=35 (entire cohort)	59.3 years, n=17

Legend: Acute inflammatory demyelinating polyneuropathy (AIDP); Acute motor axonal neuropathy (AMAN); American Diabetes Association (ADA); Cerebrospinal fluid (CSF); Charcot-Marie-Tooth Disease (CMT); Chronic inflammatory demyelinating polyneuropathy (CIPD); Electromyography (EMG); European Federation of Neurological Societies/Peripheral Nerve Society (EFNS/PNS); Guillain-Barre Syndrome (GBS); Michigan Neuropathy Screening Instrument (MNSI); National Institute of Neurological Disorders and Stroke (NINDS); Nerve conduction velocity (NCV); Not available (NA); Polyneuropathy disability score (PND); Quantitative sensory testing (QST); World Health Organization (WHO)

© 2022 Fundaun J et al. *JAMA Network Open.*

eTable 3. Summary of Biomarker Results Reported in Single Studies

Biomarker	Diagnosis	Reported results compared to controls
**Myelin associated integrin[44]**	Guillain-Barre Syndrome	Increased
**Piccolo[42]**	Guillain-Barre Syndrome	Increased
**Anti-neuroblastoma cell antibodies[54]**	Rheumatoid arthritis with peripheral neuropathy	Increased
**p75 neurotrophin receptor[34]**	Acute inflammatory demyelinating polyneuropathy	Increased
	Chronic inflammatory demyelinating polyneuropathy	Increased
	Charcot-Marie-Tooth Disease	No significant difference
**Tau[52]**	Critical illness polyneuropathy	No significant difference
**GAP-43[24]**	Diabetic neuropathy	Decreased
**BACE1[45]**	Acute inflammatory demyelinating polyneuropathy	Downregulated
	Chronic inflammatory demyelinating polyneuropathy	Downregulated
**Neurotrophin-3[31]**	Diabetic neuropathy	Decreased

© 2022 Fundaun J et al. *JAMA Network Open.*

eTable 4. Quality Assessment Using the Newcastle-Ottawa Scales

Author (year)	Selection	Comparability	Outcome	Total score
**Case-control**	**(/4)**	**(/2)**	**(/3)**	**(/9)**
Afarideh et al. (2019)[21]	4	2	1	7
Altman et al. (2020)[40]	4	2	2	8
Azoulay et al. (2020)[22]	4	2	2	8
Bischof et al. (2018)[53]	3	0	1	4
Celikbilek et al. (2014)[23]	3	2	1	6
Ghafouri-Fard et al. (2021)[45]	3	2	1	6
Hayashi et al. (2021)[46]	2	1	1	4
Kapoor et al. (2019)[48]	4	0	0	4
Kim et al. (2019)[34]	2	1	0	3
Kortvelyessy et al. (2020)[41]	2	1	1	4
Li et al. (2021)[24]	4	2	2	8
Mariotto et al. (2020)[56]	3	1	1	5
Mateos-Hernandez et al. (2016)[42]	2	0	0	2
Millere et al. (2021)[35]	3	2	1	7
Niezgoda et al. (2017)[26]	2	1	1	4
Rossor et al. (2016)[36]	4	2	1	7
Salih et al. (2000)[54]	3	1	1	5
Sandhu et al. (2008)[28]	2	0	1	3
Qiao et al. (2015)[29]	4	2	2	8
Sessa et al. (1997)[44]	3	1	1	5
Sun et al. (2018)[30]	4	2	2	8
Ticau et al. (2021)[50]	4	2	1	7
Wang et al. (2020)[38]	4	1	1	6
Wang et al. (2021)[39]	4	1	1	6
**Cross-sectional**	**(/5)**	**(/2)**	**(/3)**	**(/10)**
Li et al. (2013)[25]	4	2	3	9
Xiaowei et al. (2014)[51]	3	1	1	5
Ozuguz et al. (2016)[27]	4	2	2	8
Maia et al. (2020)[49]	4	2	3	9
Sandelius et al. (2018)[37]	3	2	2	8
Ziegler et al. (2019)[31]	4	2	1	7
Celikbilek et al. (2014)[33]	4	2	1	7
**Cohort**	**(/4)**	**(/2)**	**(/3)**	**(/9)**
Frithiof et al. (2021)[52]	4	1	1	6
Jadhav et al. (2011)[55]	2	0	2	4
Lieverloo et al. (2019)[47]	4	2	3	9
Martin-Aguilar et al. (2021)[43]	4	1	2	7
Morgenstern et al. (2021)[32]	4	2	3	9

© 2022 Fundaun J et al. *JAMA Network Open.*

eTable 5. Diagnostic Criteria and Neurofilament Light Chain Concentrations Used in the Subgroup Meta-analysis Comparing Primarily Axonal vs Demyelinating Peripheral Neuropathies

Source (author, year)	Diagnosis (number)	Diagnostic criteria	Control type (number)	Patient concentration: mean (SD)	Control concentration: mean (SD)
**Altmann et al,[40] 2020**	**GBS** Demyelinating, (n=17)<br><br>Axonal, (n=5)	Neurologic examination, Brighton criteria (levels 1 or 2), NCV	Patients without neuropathy, n=22	Demyelinating: 161.64 (124.83) pg/mL*<br><br>Axonal: 62.96 (50.25) pg/mL*	9.07 (3.24) pg/mL*
**Martin-Aguilar et al,[43] 2021**	**GBS** AMAN, (n=12)<br><br>AMSAN, (n=7)<br><br>AIDP, (n=58)	NINDS classification, Brighton diagnostic criteria, NCV	Healthy, n=53	AMAN: 386.67 (381.74) pg/mL*<br><br>AMSAN: 589.85 (1274.50) pg/mL*<br><br>AIDP: 93.57 (183.90) pg/mL*	9.67 (6.42) pg/mL*
**Sandelius et al,[37] 2018**	**CMT** CMT1 – demyelinating, (n=48)<br><br>CMT2 – axonal, (n=27)	Genetically confirmed CMT, NCV	Healthy, n=67	CMT1: 25.23 (11.80)* pg/mL<br><br>CMT2: 25.93 (12.31)* pg/mL	15.57 (7.48) ng/mL*
**Lieverloo et al,[47] 2019**	**CIDP** Induction, n=29<br><br>Maintenance, n=24<br><br>Remission, n=27	EFNS/PNS 2010 criteria, neurologic exam, NCV	Healthy, n=30	Induction: 47.33 (38.18) pg/mL*<br><br>Maintenance: 29.7 (14.06) pg/mL*<br><br>Remission: 30.67 (18.02) pg/mL*	24.03 (10.80) pg/mL*
**Hayashi et al,[46] 2021**	**CIDP**, n=11	EFNS/PNS 2010 criteria	Healthy, n=7	166.6 (337.8) pg/mL*	12.2 (6.3) pg/mL*
**Wang et al,[38] 2020**	**CMT1**, Group 1 n=20 Group 2 n=31	Clinical examination, NCV, confirmatory genetic testing, Rasch modified CMT Neuropathy and Examination Scores	Healthy, Group 1 n=20 Group 2 n=24	(NPX): Group 1: 3.60 (0.38)*<br><br>Group 2: 3.52 (0.76)*	(NPX) Control 1: 2.91 (0.43)*<br><br>Control 2: 2.84 (0.44)*
**Wang et al,[39] 2021**	**CMT1**, n=41	Clinical examination, NCV, confirmatory genetic testing,	Healthy, n=40	(NPX): 3.5 (0.60)*	(NPX): 2.83 (0.45)*

© 2022 Fundaun J et al. *JAMA Network Open.*

		Rasch modified CMT Neuropathy and Examination Scores			

Legend: Legend: Acute inflammatory demyelinating polyneuropathy (AIDP); Acute motor axonal neuropathy (AMAN); Acute motor-sensory axonal neuropathy (AMSAN); Brain derived neurotrophic factor (BDNF); Charcot-Marie-Tooth Disease (CMT); Charcot-Marie-Tooth Disease Type 1 (CMT1); Charcot-Marie-Tooth Disease Type 2 (CMT2); Chronic inflammatory demyelinating polyneuropathy (CIPD); European Federation of Neurological Societies/Peripheral Nerve Society (EFNS/PNS); Guillain-Barre Syndrome (GBS); National Institute of Neurological Disorders and Stroke (NINDS); Nerve conduction velocity (NCV); Neural cellular adhesion molecule (NCAM); Neurofilament light chain (NfL); Normalized protein expression (NPX); *indicates means estimated from graphs/figures

© 2022 Fundaun J et al. *JAMA Network Open.*

eTable 6. Descriptive Synthesis of Biomarker Diagnostic Accuracy

Study	Diagnosis	Comparison	Biomarker	Cut-off	Sensitivity & specificity	Area under curve
Bischof (2018)[53]	Vasculitic neuropathy	Healthy controls	NfL	155 pg/mL	Sensitivity 82%, specificity 100%	0.96
Millere (2021)[35]	Charcot-Marie-Tooth Disease	Healthy controls	NfL	8.9 pg/ml	Sensitivity 74%, specificity 95%	0.88 (95% CI: 0.83–0.93)
Maia (2020)[49]	hATTR amyloidosis with polyneuropathy	Cohort 1: PND 0 vs ≥1	NfL	10.6 pg/mL	Sensitivity 96.2%, specificity 93.8%	0.99
	hATTR amyloidosis with polyneuropathy	Cohort 2: PND 1 ≥ 2	NfL	75.7 pg/mL	Sensitivity 84.6%, specificity 80.0%	0.86
Sandelius (2018)[37]	Charcot-Marie-Tooth Disease	healthy controls	NfL	20 pg/mL	Sensitivity 71%, specificity 75%	0.76
Ticau (2021)[50]	hATTR amyloidosis with polyneuropathy	Healthy controls	NfL	37 pg/mL	false-positive rate 3.6%, true-positive rate 84.9%	0.96 (95% CI 0.96–0.97)
Wang (2020)[38]	Charcot-Marie-Tooth disease	Healthy controls	NfL	NA	NA	0.81 (95% CI 0.72–0.90)
			TMPRSS5	NA	NA	0.91 (95% CI 0.85–0.97)
Wang (2021)[39]	Charcot-Marie-Tooth disease	Healthy controls	NfL	NA	NA	0.80 (95% CI 0.70 - 0.90)
			TMPRSS5	NA	NA	0.87 (95% CI: 0.80-.94)
Ghafouri-Fard (2021)[45]	AIDP	healthy controls	BDNF	NA	Sensitivity 23%, Specificity 95%	0.56
	CIDP				Sensitivity 41%, Specificity 93%	0.68
Sun (2018)[30]	Diabetic neuropathy	Healthy controls	BDNF	1981.05 pg/mL	Sensitivity 98.5%, specificity 74.5%	0.93
			NGF	50.25 pg/mL	Sensitivity 96.9%, specificity 77.3%	0.93
Li (2013)[25]	Diabetic neuropathy	Diabetic without neuropathy	NSE	10.1 µg/L	Sensitivity 66.3%, specificity 72.5%	0.73 (95% CI 0.68–0.77)
Kim (2019)[34]	CIDP	CMT	p75	56.45 pg/mL	Sensitivity 92.1%, specificity 95%	0.97

© 2022 Fundaun J et al. *JAMA Network Open.*

		AMAN		103.4 pg/mL	Sensitivity 63.2%, specificity 85%	0.78
		AIDP		103.4 pg/mL	Sensitivity 84.6%, specificity 40%	0.37
	Charcot-Marie-Tooth Disease	CIDP	Neural cellular adhesion molecule	7038.7 pg/mL	Sensitivity 39.5%, specificity 100%	0.67
		AIDP		6896.1pg/mL	Sensitivity 42.1%, specificity 100%	0.85
		AMAN		3937.1 pg/mL	Sensitivity 97.4%, specificity 72.2%	0.85

Legend: Acute inflammatory demyelinating polyneuropathy (AIDP); Acute motor axonal neuropathy (AMAN); Brain derived neurotrophic factor (BDNF); Chronic inflammatory demyelinating polyneuropathy (CIDP); Confidence interval (CI); hereditary transthyretin-mediated (hATTR); Not available (NA); Nerve growth factor (NGF); Neurofilament light chain (NfL); Neuron-specific enolase (NSE); Polyneuropathy disability score (PND); Transmembrane protease serine 5 (TMPRSS5)

© 2022 Fundaun J et al. *JAMA Network Open.*

eFigure 1. Meta-analysis of Neurofilament Light Chain Comparing Axonal and Demyelinating Subtypes in Patients With Peripheral Neuropathy Compared With Controls

Source	SMD (95% CI)
**Axonal**	
Altmann et al,[40] 2020	2.57 [1.35; 3.79]
Sandelius et al[37] 2018	1.11 [0.63; 1.58]
Martín-Aguilar et al,[43] 2021	1.27 [0.60; 1.93]
Martín-Aguilar et al,[43] 2021	0.74 [0.35; 1.12]
**Total**	**1.22 [0.18; 2.27]**
**Demyelinating**	
Altmann et al,[40] 2020	1.82 [1.06; 2.58]
Hayashi et al,[46] 2021	0.55 [-0.42; 1.52]
Lieverloo et al, (induction)[47] 2019	0.68 [-0.06; 1.41]
Lieverloo et al, (maintenance)[47] 2019	0.42 [-0.33; 1.16]
Lieverloo et al, (remission)[47] 2019	0.39 [-0.34; 1.13]
Sandelius et al[37] 2018	1.01 [0.61; 1.40]
Wang et al, (cohort 1)[38] 2020	1.67 [0.94; 2.40]
Wang et al, (cohort 2)[38] 2020	1.05 [0.48; 1.62]
Wang et al,[39] 2021	1.25 [0.77; 1.73]
Martín-Aguilar et al,[43] 2021	0.63 [0.24; 1.01]
**Total**	**0.95 [0.62; 1.29]**
**Total**	**1.01 [0.73; 1.29]**

Heterogeneity: $\chi^2_{13} = 27.70$ (P = .010), $I^2 = 53\%$
Test for subgroup differences: P = .45

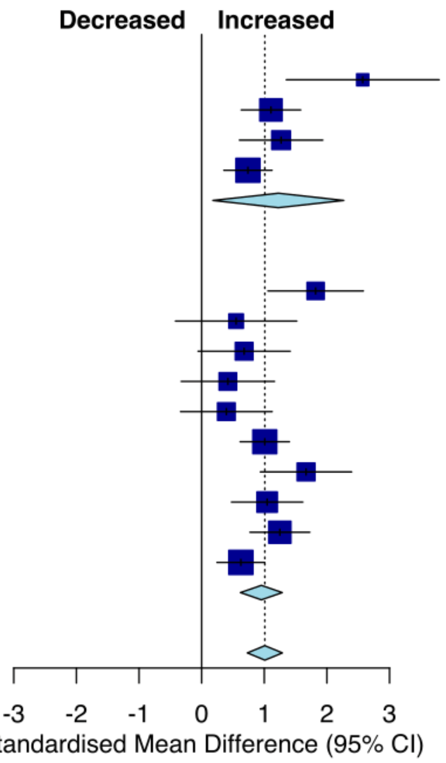

© 2022 Fundaun J et al. *JAMA Network Open.*

eFigure 2. Bulk Tissue Gene Expression Profile for Neurofilament Light Chain According to GTEx Portal (accessed on June 6, 2022)[60]

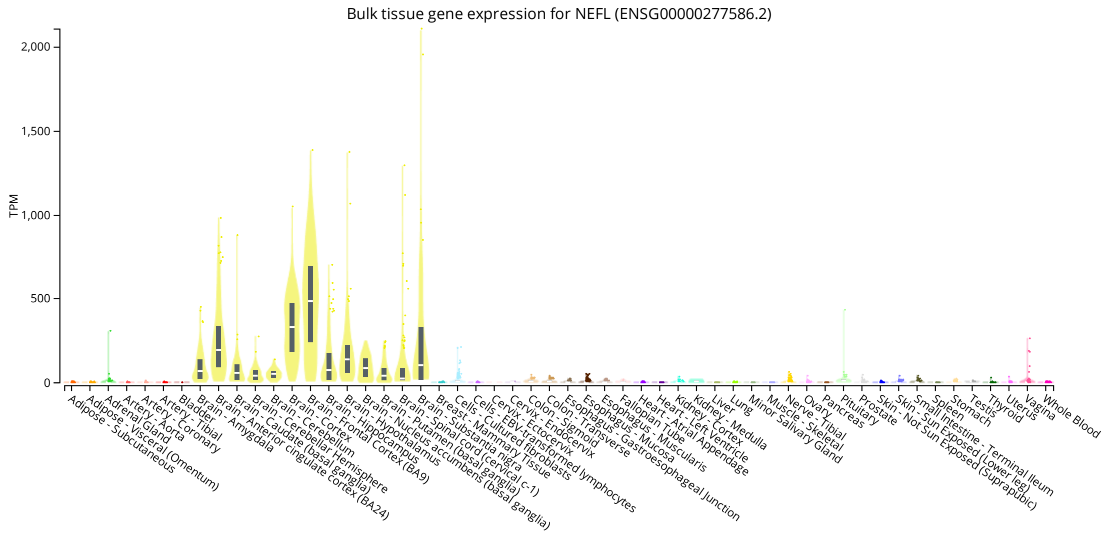

© 2022 Fundaun J et al. *JAMA Network Open.*
