# Supplementary material for: Types and Concentrations of Blood-Based Biomarkers in Adults With Peripheral Neuropathies: A Systematic Review and Meta-analysis
Source: JAMA Netw Open. 2022 Dec 27;5(12):e2248593. doi: 10.1001/jamanetworkopen.2022.48593 (PMC9857490; doi:10.1001/jamanetworkopen.2022.48593)
Supplement: Supplement 2. — Data Sharing Statement [file jamanetwopen-e2248593-s002.pdf]

Data Sharing Statement

Fundaun. Types and Concentrations of Blood-Based Biomarkers in Adults With Peripheral Neuropathies. *JAMA Netw Open*. Published December 27, 2022.
doi:10.1001/jamanetworkopen.2022.48593

Data
Data available: No

Additional Information
Explanation for why data not available: As this is a meta-analysis of previous published studies, our extracted data will be shared upon reasonable request.
